# Abductor pollicis longus tendon division with swan neck thumb deformity

**DOI:** 10.1007/s11751-012-0141-8

**Published:** 2012-07-24

**Authors:** Balaji Zacharia, Kishore Puthezhath

**Affiliations:** 1 Department of Orthopaedics, Government Medical College, Calicut, India; 2 Department of Orthopedics, Amala Institute of Medical Sciences, Thrissur, India; 3 33/6220C, Kakkathottam House, Near Government Leprosy Hospital, Chevayoor PO, Calicut, 673017 Kerala India

**Keywords:** Isolated division, Abductor pollicis longus tendon, Swan neck thumb deformity

## Abstract

Swan neck thumb deformity can be caused by osteoarthritis, rheumatoid arthritis, systemic lupus erythematosus, tendon transfers and paralytic diseases. Abductor pollicis longus is one of the major stabilizing tendon of the carpometacarpal joint of thumb. To the best of our knowledge, swan neck thumb deformity owing to division of abductor pollicis longus tendon is rare. In this article, we describe a case of isolated division of abductor pollicis longus tendon presenting with swan-neck deformity of thumb and discuss the mechanism, management and outcome. The patient was treated by repair of the divided tendon using palmaris longus tendon graft. At approximately 107 weeks following treatment, the patient was having full range of thumb movement and the deformity completely disappeared. We also describe the unusual mechanism whereby an isolated division of abductor pollicis longus tendon results in swan neck thumb deformity. *Level of clinical evidence* IV.

## Introduction

Swan neck thumb (SNT) deformity is characterized by flexion at the interphalangeal joint and extension at the metacarpophalangeal joint. SNT deformity is not uncommon, and cases of SNT associated with osteoarthritis, rheumatoid arthritis, systemic lupus erythematosus, paralytic diseases and Flexor digitorum superficialis tendon transfer for intrinsic replacement have been reported [1–4]; however, to the best of our knowledge, SNT deformity as a result of isolated complete division of abductor pollicis longus tendon (APL) has heretofore not been reported in the biomedical literature.

## Case report

**Fig. 1:**
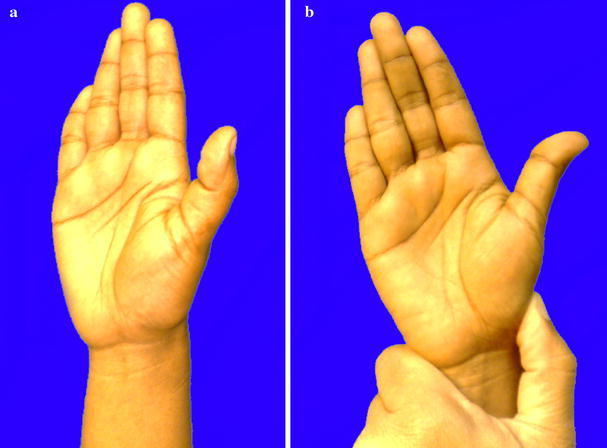
**a**, **b** Photographs showing subluxation of carpometacarpal joint of the right thumb and on reducing the subluxation, the deformity of the thumb gets corrected

**Fig. 2:**
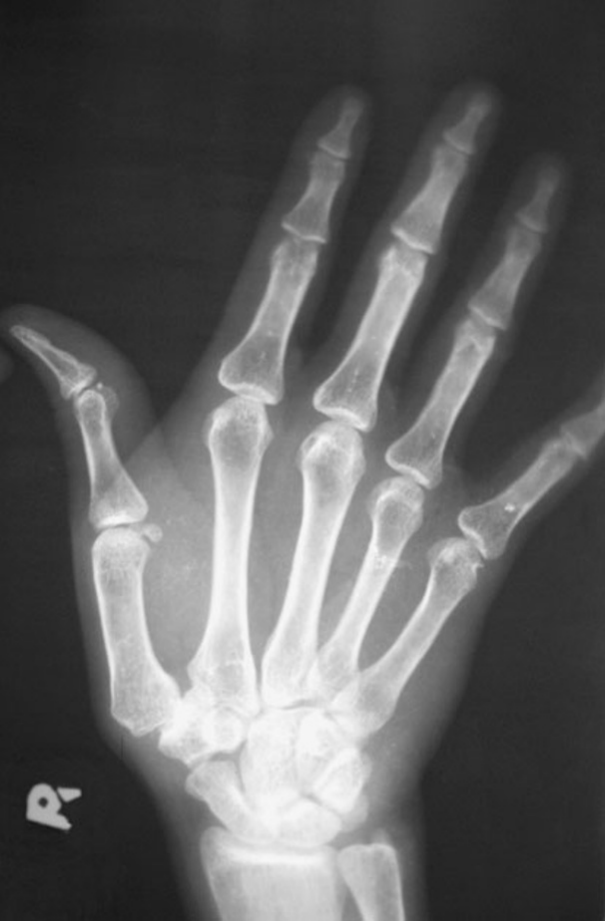
Preoperative radiograph showing subluxation of carpometacarpal joint of the right thumb

A 23-year-old woman had an accidental stab injury to her right wrist with a knife while doing some household work. The wound healed without any treatment. Approximately 1 year later, the patient gradually developed pain in the right wrist. She was initially treated with non-steroidal anti-inflammatory analgesics. The patient was referred to Government Medical College, Calicut due to persistent right wrist pain and was subsequently evaluated approximately 60 weeks after the injury. The patient had a small scar, which was not easily noticeable, over the dorsolateral aspect of right wrist and tenderness over the styloid process of the right radius. Right thumb was kept with flexion at the interphalangeal joint and extension at the metacarpophalangeal joint. There was subluxation of carpometacarpal joint of the right thumb, and on reducing the subluxation, the deformity of the thumb gets corrected (Fig. 1a, b). The patient’s right abductor pollicis longus was not acting. A radiogramme of the right hand showed subluxation of the carpometacarpal joint of the thumb (Fig. 2).

**Fig. 3:**
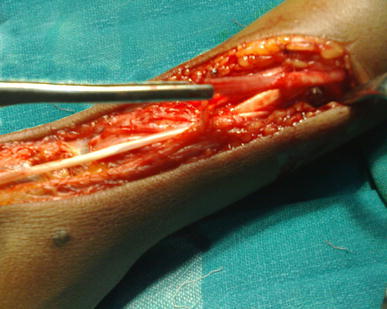
Operative photograph showing complete isolated division of the right abductor pollicis longus tendon

**Fig. 4:**
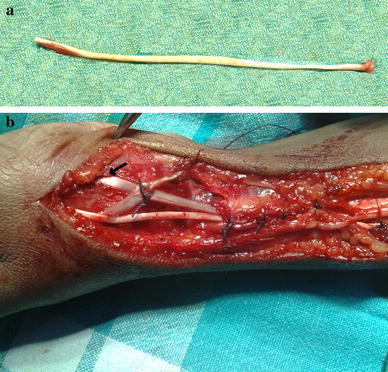
**a** Palmaris longus tendon graft taken from the ipsilateral side. **b** Reconstruction by approximating the proximal end of abductor pollicis tendon to the graft and distally through a tunnel in the base of first metacarpal (*black arrow*)

Based on the history of injury to the right wrist and clinical examination results, it was decided that best treatment option for this patient was surgical exploration and stabilization of right carpometacarpal joint of the thumb. Exploration was thereafter performed under axillary block anaesthesia, which was established with the aid of a nerve locator. A 15-cm-long lateral incision was used to expose the first extensor compartment, the radial styloid and the carpometacarpal joint of the thumb. There was a complete isolated division of the right abductor pollicis longus tendon (Fig. 3). Per-operatively the capsule of the carpometacarpal joint of the thumb did not show any tear, and there was no avulsion of the abductor pollicis longus tendon from its insertion. Due to retraction of the cut ends beyond approximation, the divided tendon was reconstructed using a palmaris longus tendon graft taken from the ipsilateral side approximating the proximal end of abductor pollicis tendon to the graft and distally through a tunnel in the base of first metacarpal (Fig. 4a, b).

Following the operation, the right hand was immobilized in a short-arm cast for about 4 weeks, with thumb in an abducted and extended position for keeping the first carpometacarpal joint in reduced position. Overall, the postoperative course was uneventful with the exception of mild stiffness of the right hand and fingers that subsided with subsequent mobilization and a short course of physiotherapy. At 107-week follow-up, the patient had long before returned to normal routine work, regained full mobility of thumb and had the deformity of thumb completely disappeared.

## Discussion

APL originates from the posterior surface of ulna and interosseus membrane and attaches to the radial side of the base of first metacarpal bone. It abducts and extends carpometacarpal joint of the thumb. From experiments with tendon movements, there are strong indications that the deep division, particularly, has a stabilizing function on the basal joint of the thumb [5]. The oblique fibres of adductor pollicis originate from the capitate and base of the second metacarpal, and its transverse head from palmar surface of third metacarpal. Adductor pollicis gets attached to the base of proximal phalanx of thumb and to extensor expansion. Adductor pollicis adducts carpometacarpal joint of the thumb.

The trapeziometacarpal joint is a loose-fitting saddle joint which has double concave surfaces permitting great freedom of movement in three planes, enabling retroposition and opposition, adduction and abduction, and circumduction. Because of the laxity of the joint capsule in young Asian female population and the incongruity of the joint surfaces, slipping occurs at extreme adduction and opposition. Trapeziometacarpal joint instability affects the moment arms of thumb motor tendons restricting the movement at the base of the thumb [6]. Compensatory movements occur in the distal joints to provide thumb function. Instability and deformity, such as the swan-neck deformity, may result from these compensatory changes.

In the case that we describe in this report, the patient had a neglected, trivial looking cut injury of wrist. We believe that the cut injury may have disrupted the APL tendon injury that was identified intraoperatively, thereby causing CMC subluxation and subsequent SNT deformity. Isolated division of APL tendon results in adduction of first metacarpal due to over action of adductor pollicis. This results in subluxation of first metacarpal dorsally and laterally. With subluxation of CMC joint and relative adduction of first metacarpal bone, the normal moment arm of the extensor pollicis longus motor tendon is decreased and movement at the base of the thumb is compromised. The result is over action of extensor pollicis brevis causing hyperextension and further adduction of the first metacarpal. The more the adduction contracture of the metacarpal, the greater is the tendency for the metacarpophalangeal joint to hyperextend and the thumb ray to collapse [7, 8]. A vicious circle is, therefore, established. Further, over action of long flexor of thumb and underaction of long extensor cause flexion of the proximal interphalangeal joint, resulting in swan neck thumb deformity. We believe that this mechanism of injury causing SNT deformity is unusual and that the patient in this article represents the first such case described in the literature.

**Fig. 5:**
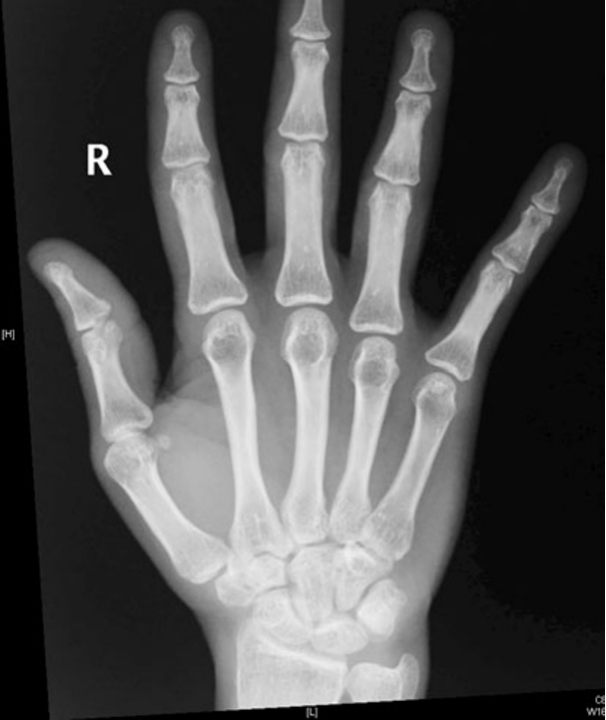
Postoperative radiograph showing complete reduction in carpometacarpal joint of right thumb

Anatomical reduction in the CMC joint and stabilization has been shown to improve the clinical results in SNT deformity due to other causes [6, 9]. In the present case, an exploration was undertaken in an effort to facilitate the reconstruction of APL using Palmaris longus tendon graft. The 4-week course of immobilization that we pursued had previously been suggested for extensor tendon injury. Our main reason for pursuing exploration was to restore the anatomical alignment of the joint and to repair or reconstruct the APL tendon which was clinically not acting, with the understanding that such intervention is critical to normal thumb biomechanics. Once APL was found disrupted beyond repair, our choice of Palmaris longus tendon for the reconstruction of APL instead of arthrodesis and volar capsulorrhaphy was based on the concept that useful movement is retained and the metacarpophalangeal joint of the thumb is exposed by nature of its function to more extension stress than are the metacarpophalangeal joints of the fingers [8, 9]. Despite developing CMC joint subluxation and SNT deformity, stabilization of CMC joint by APL reconstruction resulted in complete relief of symptoms and the deformity (Fig. 5).

In conclusion, isolated APL division causing swan neck thumb deformity is a relatively rare injury. Furthermore, isolated APL division may predispose to subluxation or dislocation of the carpometacarpal joint of the thumb. Still further, we feel that surgeons should carefully assess the integrity of APL whenever SNT deformity is encountered. Finally, because of the risk of developing CMC dislocation and posttraumatic arthrosis, surgical exploration and stabilization of the CMC joint either by repair or by reconstruction of the APL seems to be beneficial in such patients with SNT deformity due to APL division.
